# Diffusion Processes and Drug Release: Capsaicinoids - Loaded Poly (ε-caprolactone) Microparticles

**DOI:** 10.1371/journal.pone.0157662

**Published:** 2016-06-16

**Authors:** E. K. Lenzi, A. Novatski, P. V. Farago, M. A. Almeida, S. F. Zawadzki, R. Menechini Neto

**Affiliations:** 1 Departamento de Física, Universidade Estadual de Ponta Grossa, Ponta Grossa, 84030–900, Brazil; 2 Departamento de Ciências Farmacêuticas, Universidade Estadual de Ponta Grossa,Ponta Grossa, 84030–900, Brazil; 3 Departamento Química, Universidade Federal de Curitiba, Curitiba, 81531–980, Brazil; Tsinghua University, CHINA

## Abstract

We present a generalmodel based on fractional diffusion equation coupled with a kinetic equation through the boundary condition. It covers several scenarios that may be characterized by usual or anomalous diffusion or present relaxation processes on the surface with non-Debye characteristics. A particular case of this model is used to investigate the experimental data obtained from the drug release of the capsaicinoids-loaded Poly (ε-caprolactone) microparticles. These considerations lead us to a good agreement with experimental data and to the conjecture that the burst effect, i.e., an initial large bolus of drug is released before the release rate reaches a stable profile, may be related to an anomalous diffusion manifested by the system.

## Introduction

Diffusion is one of the most important phenomena present in all natural science, from biology to physics. In biological contexts (living organisms)[1–5], it may manifest different characteristics during the spreading of the system and, in several situations, it is not Markovian, i.e., the mean square displacement has a nonlinear time dependence and presents different regimes. This unusual behavior has been related to the complexity of these systems (e.g. morphology) and, consequently, to the process presented in these scenarios[6–8]. Similar features are also observed in pharmacokinetics, where understanding the controlled drug delivery processes plays an important role for obtaining a suitable treatment for patients, minimizing the undesired effects of the drug in the organism. Because of that, several mathematical models have been used to face the point, taking into account the drug release *in vitro*, where diffusion and kinetic processes are investigated[9, 10].

The first model, called zero-order kinetics, is based on the release of an active substance from the pharmaceutical dosage form as a function of time. This model can be expressed as *C*_*t*_ / *C*_∞_ = *α*_0_*t* + *b* [11, 12] where *b* is the initial quantity of drug in bulk due to an immediate process releasing. Another model, proposed by Higuchi (1961)[13], leads to the following equation: $C_{t}/C_{\infty }=\alpha_{H}\sqrt{t}+b$, where *α*_*H*_ depends on drug solubility and on the characteristics of the polymeric matrix. This model is directly related to the Fick´s law and has been successfully applied in situations where the swelling process is absent. Korsmeyer et al.[9, 14]extended both models by proposing the semi-empirical equation *C*_*t*_ / *C*_∞_ = *αt*^*n*^ + *b*, where α is a kinetic constant related to the structural and geometric characteristic of the system. Depending on *n* value (also connected to geometric form), we may have a diffusive process (*n≈0*.*45*), swelling (*n>0*.*89*), or anomalous behavior (*0*.*45<n<0*.*89*), i.e., a blend of diffusion and swelling. This equation is used, in general, to analyze the initial stage of the drug release process up to 60%, regardless of the geometric shape. Therefore, it is possible to obtain information (depending on*n* value) on the process manifested during the drug release, for both processes, diffusion and swelling, which may occur simultaneously. In fact, the power law can be considered as an extension that enables us to observe and quantify the superposition of two apparently independent mechanisms of drug transport[9, 15]. It also describes in many cases dynamic swelling and drug release from the matrix, regardless of the constitutive equation type of coupling.

**Fig 1:**
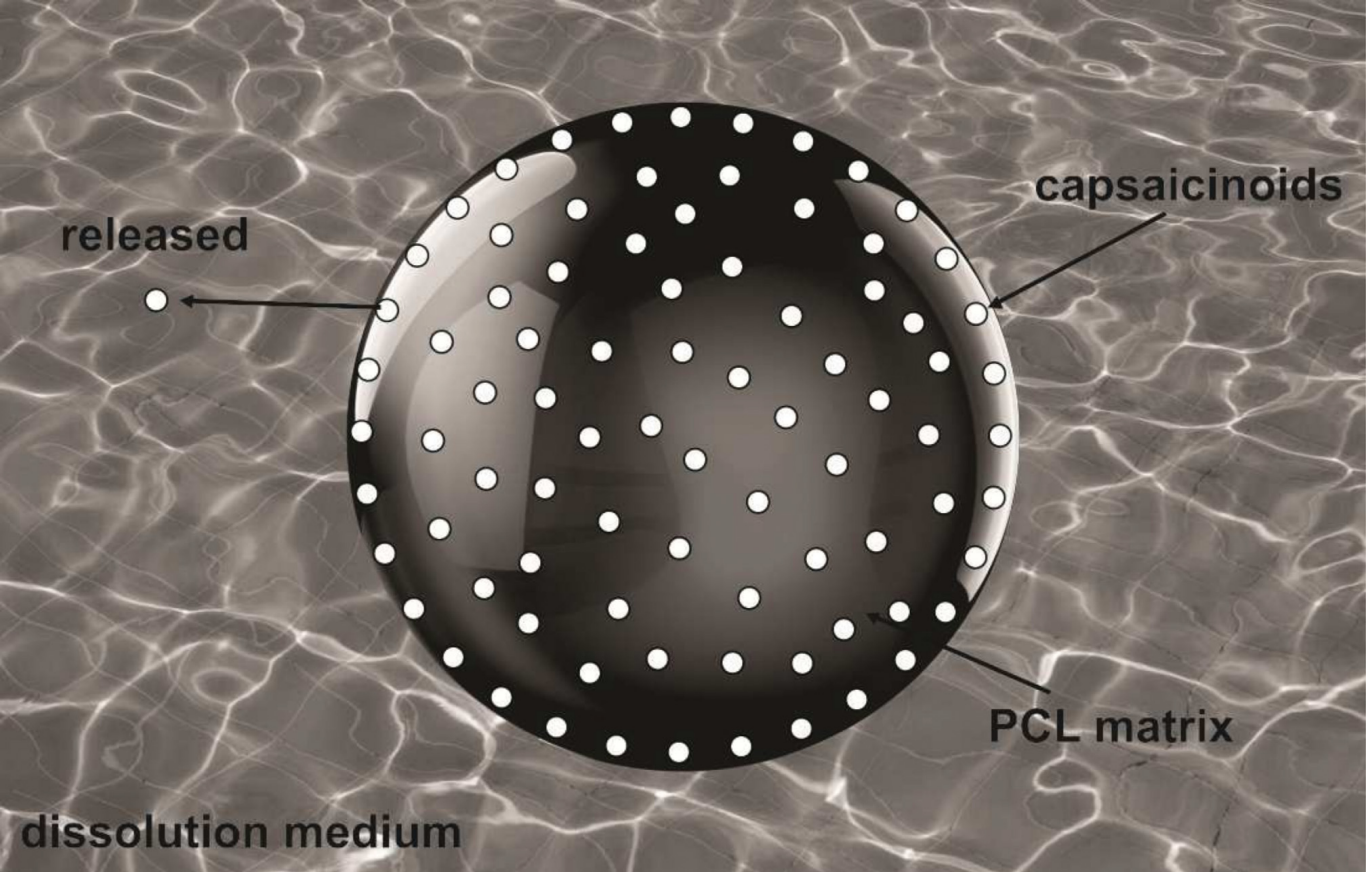
Release process. Illustration of the capsaicinoids releasing process from the surface of PCL microparticles to bulk.

In this study, we propose and analyze some aspects of a phenomenological model to drug release. A particular case of this model is applied to the analysis of experimental data of capsaicinoids–loaded PCL microparticles[16], where a complete understanding on the drug release mechanism is still lacking. Despite the pharmacological properties of capsaicinoids, e.g. treatment of chronic pain, obesity, and cancer, these drugs are irritating substances that produce burning on skin and mucous membranes in low concentrations[17].In this sense, a polymeric system as poly (ε–caprolactone) microparticles can provide both an initial effect through rapid drug release and a long-term one, by means of a controlled process. This scenario is illustrated in Fig 1, where the drug is desorbed from a polymeric surface with spherical symmetry.In particular, we have obtained a good agreement between the experimental data and the model. This feature provides evidence that diffusion and kinetic processes on surface describe in a suitable way the polymeric system when they are treated simultaneously.

## Diffusion and Surface Dynamics

### The phenomenological model

The model considers the diffusive and the kinetic processes simultaneously. They are coupled through the boundary conditions, which connect processes on the surface of microparticles with the diffusion of the substance in the bulk. In this perspective, the kinetic and the diffusion processes are mutually involved and one may influence on the other, in contrast to the previous models. It is based on the following fractional diffusion equation of distributed order[18]:

∂∂tCbulk(r,t)=K1r2∂∂rr2{∫01dγ p(γ)D0t1−γ(∂∂rCbulk(r,t))} (1)

Itmayalso be obtained, from the phenomenological point of view, from the continuity equation $\partial_{t}C_{bulk}+\nabla \cdot \vec{J}=0$ with the current density given by

J→(r,t)=K∫01dγ p(γ)D0t1−γ(∂∂rCbulk(r,t))r^. (2)

Eq (1) is subjected to the boundary conditions:

$$\;\oint \vec{J}(R,t)\cdot d\vec{S}=\frac{d}{dt}\;C_{surf}(t),$$ (3)

4πR2K∫01dγ p(γ)D0t1−γ∂∂rCbulk(r,t)|r=R=ddt Csurf(t), (4)

and

$${\frac{\partial }{\partial r}C_{bulk}(r,t)|}_{r=\infty }=0\;.$$ (5)

In Eq (1), *C*_*bulk*_(*r*,*t*) is the concentration of the substance in bulk, K is the diffusion coefficient,D0t1−γ(⋯) represents the Riemann—Liouville fractional differential operator[19, 20], and *p*(*γ*) is a distribution of *γ*. It recovers the usual diffusion equation for *p*(*γ*) = *δ*(*γ*−1) and enables us to investigate processes that are not suitably described in terms of Fick’s law. In particular,the fractional diffusion equations[21, 22] have been used to investigate several situations such as tumor[23], electrical response[24], solute transport[25] and chemotaxis[26]. One of the most important characteristics of these phenomena related to anomalous diffusion is the nonlinear time dependence. It is manifested by the mean square displacement, i.e.,$\;\langle {\left(r-\langle r\rangle \right)}^{2}\rangle \sim t^{\alpha }$ where *α<1* and *α>1* correspond to sub- or superdiffusion, respectively. This way, from the mathematical point of view, Eq (1) may describe the bulk particles in a general scenario; it allows us to work on several cases in a unified way[27, 28]. These cases can be connected to usual or anomalous diffusion. It is also important to mention that Eq (1) may also describe situations characterized by different diffusive regimes[29, 30], depending on the choice of *p*(*γ*).In Eq (4), *C*_*surf*_ (*t*) gives the quantity of substance present on the surface of the spherical region of radius R. Therefore, Eq (4) connects the flux (current density) and the variation of the substance present on the spherical surface.For *C*_*surf*_ (*t*), we consider the following kinetic equation:

$$\frac{d}{dt}C_{surf}(t)=\int_{0}^{t}dt^{\prime }k_{s}(t-t^{\prime })C_{bulk}(R,t^{\prime })-\int_{0}^{t}k_{d}(t-t^{\prime })C_{surf}(t^{\prime })dt^{\prime }$$ (6)

to describe the process on the surface of the polymeric matrix during drug release. In Eq (6), the first term is connected to the sorption of the substance from bulk to the surface and the second one gives the desorption rate of the substance (drug) from the surface to bulk. It is interesting to remark that *k*_*s*_(*t*) = (*k*/*τ*)*δ*(*t*/*τ*) leads us to a thickness characterized by *k*, defining a characteristic length of the interaction between surface and bulk. This way, *k*_*s*_(*t*) represents how the interaction between the particles happens[31], i.e., it may be connected to the characteristics of the surface. Therefore, the model covers several scenarios that may be characterized by usual or anomalous diffusion or present relaxation processes on the surface with non-Debye characteristics. It is also important to notice that the values of the parameters or the functions present in this model are fixed by experimental context. In addition, Eq (6) has as particular case the one-dimension situations worked out in Refs.[31, 32], where the memory effect in the kinetic equation is only considered in desorption term.

### Predictions

Our attention is now focused on the time—dependent solutions of Eq (1). To face this problem, we use the Laplace transform ($\;L\{C_{bulk}(r,t)\}={\bar{C}}_{bulk}(r,p)$ and $\;L^{-1}\{{\bar{C}}_{bulk}(r,p)\}=C_{bulk}(r,t)$) and the Green function approach[33]. We also consider that the initial condition is given by *C*_*bulk*_(*r*,0) = *φ*(*r*) and *C*_*surf*_ (0) = *C*, to analyze a general situation characterized by the presence of the substance in bulk or on the surface, or partial quantities in both. Applying the Laplace transform, the previous equation for *C*_*bulk*_(*r,t*) can be rewritten as

$$\bar{I}(p)K\nabla^{2}{\bar{C}}_{bulk}(r,p)=s{\bar{C}}_{bulk}(r,p)-{\bar{C}}_{bulk}(r,0)$$ (7)

with $\bar{I}(p)=\int_{0}^{1}p(\gamma )p^{1-\gamma }d\gamma$. The boundary conditions, in the Laplace space, are given by

$$4\pi R^{2}{\bar{I}(p)K\frac{\partial }{\partial r}{\bar{C}}_{bulk}(r,p)|}_{r=R}=\frac{p{\bar{k}}_{s}(p)}{p+{\bar{k}}_{d}(p)}{\bar{C}}_{bulk}(R,p)-\frac{{\bar{k}}_{d}(p)}{p+{\bar{k}}_{d}(p)}C\;,$$ (8)

$${\frac{\partial }{\partial r}{\bar{C}}_{bulk}(r,p)|}_{r=\infty }=0\text{ .}$$ (9)

Using the Green function approach, the solution of Eq (5) subjected to Eqs (6) and (7) can be formally written as

$${\bar{C}}_{bulk}(r^{\prime },p)=-4\pi \int_{0}^{\infty }drr^{2}\varphi (r)\bar{G}(r,r^{\prime };p)-\frac{{\bar{k}}_{d}(p)C}{s+{\bar{k}}_{d}(p)}\bar{G}(R,r^{\prime };p)\;,$$ (10)

with the Green function obtained from the equation

$$\bar{I}(p)K\nabla^{2}\bar{G}(r,r^{\prime };p)-p\bar{G}(r,r^{\prime };p)=\frac{1}{4\pi {r^{\prime }}^{2}}\delta (r-r^{\prime })\;,$$ (11)

and subjected to the boundary conditions

$$4\pi R^{2}{\bar{I}(p)K\frac{\partial }{\partial r}\bar{G}(r,r^{\prime };p)|}_{r=R}=\frac{p{\bar{k}}_{s}(p)}{p+{\bar{k}}_{d}(p)}\bar{G}(R,r^{\prime };p)\;,$$ (12)

$${\frac{\partial }{\partial r}\bar{G}(r,r^{\prime };p)|}_{r=\infty }=\;0.$$ (13)

By solving Eq (11), we can obtain the Green function, given by

$$\begin{array}{ll} \bar{G}(r,r^{\prime };p)= & -\frac{1}{8\pi rr^{\prime }\sqrt{p\bar{I}(p)K}}\left(e^{-\sqrt{\frac{p}{\bar{I}(p)K}}|r+r^{\prime }-2R|}-e^{-\sqrt{\frac{p}{\bar{I}(p)K}}|r-r^{\prime }|}\right)\\ & -\frac{1}{rr^{\prime }}\frac{R^{2}(p+{\bar{k}}_{d}(p))e^{-\sqrt{\frac{p}{\bar{I}(p)K}}|r+r^{\prime }-2R|}}{4\pi \bar{I}(p)KR(p+{\bar{k}}_{d}(p))\left(R\sqrt{p/\left(\bar{I}(p)K\right)}+1\right)+p{\bar{k}}_{s}(p)}\; \end{array}$$ (14)

In Eq (14), the last term is connected to the sorption—desorption process of the substance from surface to bulk, i.e., it contains the dynamic of the interaction between surface and bulk, by means of ${\bar{k}}_{d}(s)$ and ${\bar{k}}_{s}(p)$. By using these results, it is also possible to find the survival probability,$S(t)=4\pi \int_{R}^{\infty }dr^{\prime }{r^{\prime }}^{2}\rho (r^{\prime },t)$, which gives the quantity of substance present in bulk as a function of time. After some calculations, we have that

$$\begin{array}{l} \bar{S}(p)=4\pi \frac{1}{s}\int_{R}^{\infty }d\tilde{r}\tilde{r}\varphi (\tilde{r})\left(\tilde{r}-Re^{-\sqrt{\frac{p}{\bar{I}(p)K}}(\tilde{r}-R)}\right)\;\\ +\frac{4\pi R\left(\bar{I}(p)K/p\right)\left(R\sqrt{p/\left(\bar{I}(p)K\right)}+1\right)}{4\pi \bar{I}(p)RK(p+{\bar{k}}_{d}(p))\left(R\sqrt{p/\left(\bar{I}(p)K\right)}+1\right)+p{\bar{k}}_{s}(p)}\\ \times \left({\bar{k}}_{d}(p)C+4\pi (p+{\bar{k}}_{d}(p))R\int_{R}^{\infty }d\tilde{r}\tilde{r}\varphi (\tilde{r})e^{-\sqrt{\frac{p}{\bar{I}(p)K}}(\tilde{r}-R)}\right) \end{array}$$ (15)

**Fig 2:**
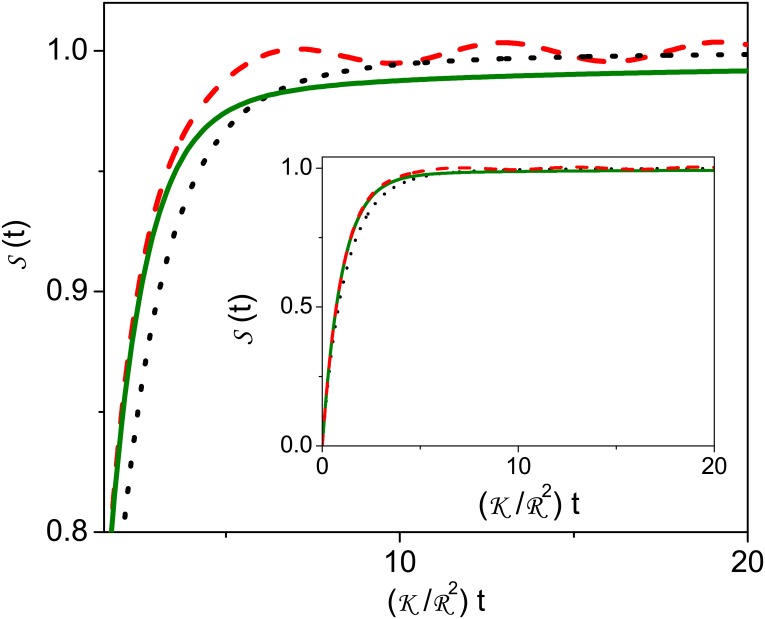
Illustration of a desorption process. This figure illustrates a desorption process characterized by the initial condition *C*_*bulk*_(*r*,0) = 0 and *C*_*surf*_(0) = 1 with *α* = 1, ${\bar{k}}_{d}(p)=k_{d,1}$, $k_{d,1}=K/R^{2}$, and *k*_*s*,1_ = *R*^3^*k*_*d*,1_. The black dotted line is the case ${\bar{k}}_{s}(p)=5k_{s,1}$. The green straight line is the case ${\bar{k}}_{s}(p)=\left({\bar{k}}_{s,1}/5\right)\left(1+4/\sqrt{p}\right)$. The red dashed line is the case ${\bar{k}}_{s}(p)=\left({\bar{k}}_{s,1}/5\right)\left(4+1/(1+p^{2})\right).$

This equation can manifest different diffusive behaviors depending on the choice of ${\bar{k}}_{d}(p)$, $\bar{I}(p)$, and ${\bar{k}}_{s}(p)$. Eqs (10), (14) and (15) represent a general solution for a scenario where sorption—desorption is present. Fig 2 illustrates the behavior of Eq (15) for different choices of ${\bar{k}}_{s}(p)$. In particular, depending on the behavior of ${\bar{k}}_{s}(p)$ the desorption process may manifest oscillations as illustrated in this figure for the case ${\bar{k}}_{s}(p)=\left({\bar{k}}_{s,1}/5\right)\left(4+1/(1+p^{2})\right).$

## Experimental Procedure and Results

**Fig 3:**
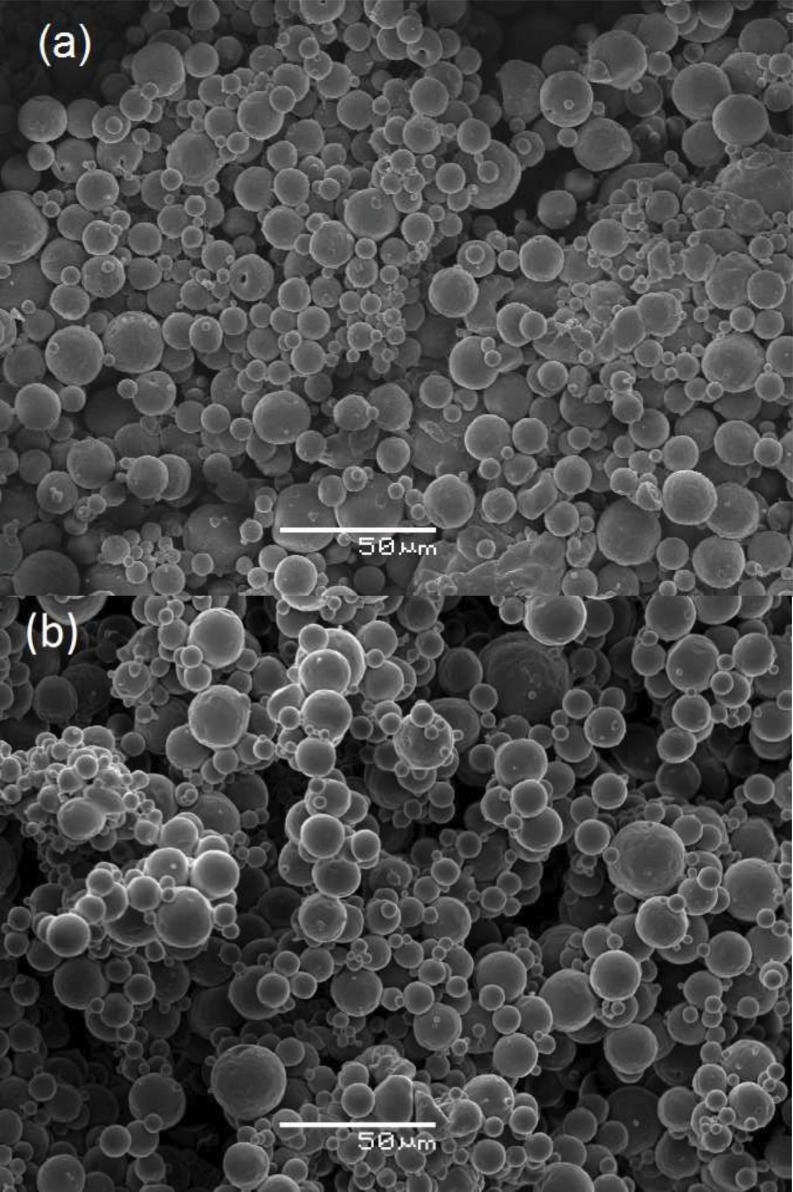
Scanning electron microscopy. Scanning electron micrographs of PCL microparticles: (a) MC3 and (b) MC5 (magnification:500x).

The simple emulsion/solvent evaporation method was performed to obtain capsaicinoids-loaded PCL microparticles as described in reference[16]. In the present work, we used the formulations containing 3 and 5% (w/w) of capsaicinoids (capsaicin and dihydrocapsaicin), named MC3 and MC5, respectively. To analyze the morphology and surface of these samples, we performed the scanning electron microscopy (SEM). To apply this technique, we used JSM-636-LV equipment, at an accelerating voltage of 10 kV with 500x of magnification. The particle size was measured by laser diffraction spectrometry in a Cilas 920 L-apparatus. The details of SEM and particle size determination can also be found in reference[16]. We show in Fig 3 the spherical morphology of MC3 (2a) and MC5 (2b) samples without porous presence on the surface. It is worth to mention that particle size is required for comparison between experimental data and model prediction. In this sense,R represents radius average of spherical particles present in experiment and the boundary condition of the model, i.e., Eqs 2 and 12 are fixed on this surface. Besides that, we consider that the drug release process in each spherical particle is independent of each other.

To investigate the drug release process, dissolution experiments were performed with formulations MC3 and MC5 using a Nova Etica 299–6 ATTS dissolution tester equipped with paddles. The system was kept at a thermostatically controlled temperature of 37 ± 0.5°C and stirred at 75 rpm. The dissolution medium chosen was degassed phosphate buffer (50 mM, pH 6.8, 900 mL) as previously reported[16]. At fixed time intervals, samples were collected, filtered (0.45 *μ*m pore size) and analyzed spectrophotometrically at 210 nm using a Genesys 10S spectrophotometer.

**Fig 4:**
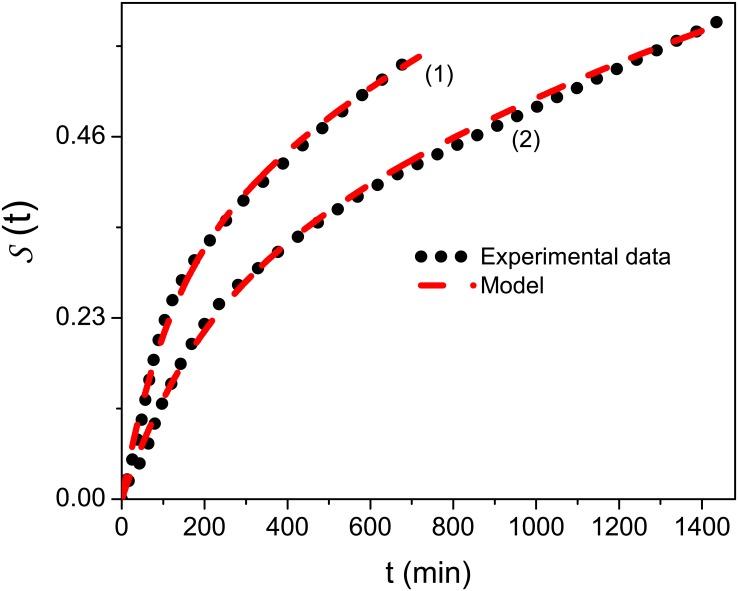
Model and experimental data. This figure compares the proposed model with experimental data present in Ref.[16], where (1) corresponds to MC5 and (2) to MC3. We have used in both cases *k*_*s*_(*p*) = 0 and *k*_*d*_(*p*)/*p*^2^ = (*p+k*_1_)/(*k*_2_*p+k*_3_). For formulation MC5 (1), we used *k*_1_ = 5.71×10^−5^*s*^−1^, *k*_2_ = 0.99*s*, and *k*_3_ = 0.23. For MC3 (2), we used *k*_1_ = 9.6×10^−5^*s*^−1^, *k*_2_ = 0.99*s*, and *k*_3_ = 0.347.

The dissolution value was obtained from the amount of drug released. A correction factor was applied to the cumulative dilution caused by replacement of the sample with an equal volume of fresh medium. Fig 4 depicts the results of *in vitro* drug release experiments. Formulation MC3 (2) showed a slower release of capsaicinoids in comparison to MC5 (1). It demonstrates that PCL content plays a relevant role on the delay of drug dissolution.

Fig 4 shows the agreement between the experimental data and predictions, with *R*^2^ = 0.997 of confidence, obtained from Eq (13), with the initial condition *C*_*bulk*_(*r*,0) = 0 and *C*_*surf*_ (0) = *C*. This scenario characterizes the drug release and diffusion from the polymer surface to bulk, in contrast to previous models[8, 10, 34]where the processes is evaluated inside the polymeric system. This feature is evidence that diffusion and kinetic processes connected to drug releasing may lead us to a suitable result when they are simultaneously considered to analyze the experimental data.

**Fig 5:**
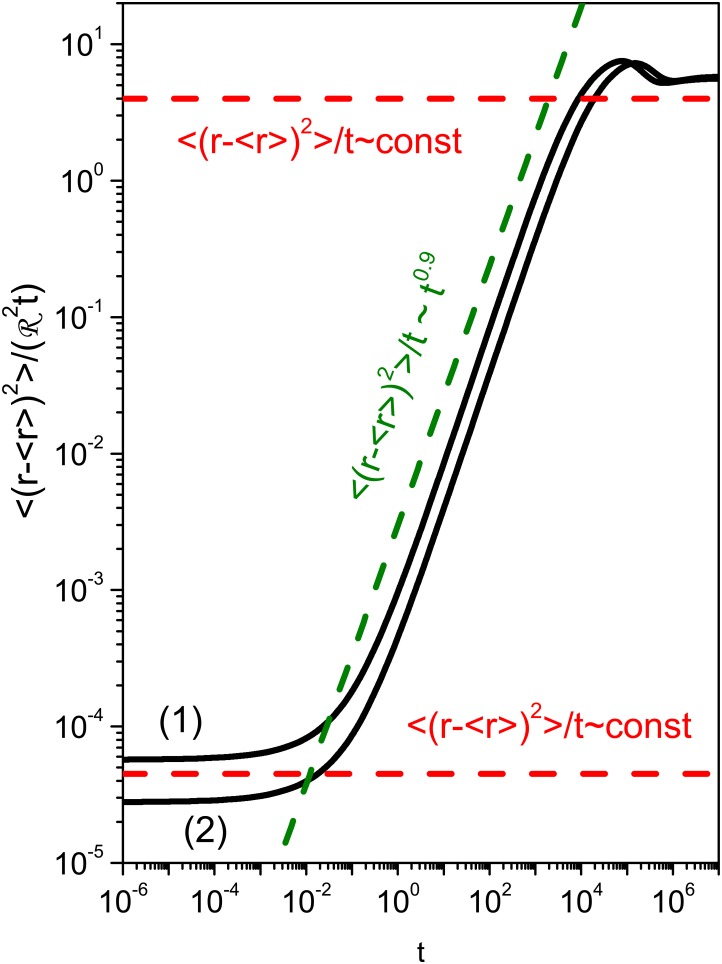
Behavior of the mean square displacement. The time dependent behavior of the mean square displacement for the situations analyzed in Fig (4) is shown. We observe that the diffusion is usual for short and long times in both cases. For intermediate times, the diffusive regime is different from the usual one, with superdiffusive characteristics. These regimes are evidenced by the red and green dashed lines. In this figure, we used $K\sim 10^{-10}\;m^{2}s^{-1}$, $R_{1}\sim 9\times 10^{-6}\;m$, $R_{2}\sim 9.3\times 10^{-6}\;m$, and *p*(*γ*) = *δ*(*γ*−1).

The behavior exhibited by the mean square displacement for the situations discussed in Fig 4 shows different diffusive regimes. They are observed for intermediate times and are directly related to the coupling considered between the surface effects and the bulk dynamics, where one modifies the other. The anomalous regime may be connected to the burst effect, which occurs during drug release. This way, we conjecture that the high initial rates of drug delivery may lead us to an unusual diffusion process after some time, which corresponds to the intermediate part of Fig 5 with a nonlinear time dependence for $\langle {\left(r-\langle r\rangle \right)}^{2}\rangle$[31]. Unusual behavior for the mean square displacement was also reported in Refs.[35, 36], by taking into account the cylindrical symmetry and surface diffusion with the bulk dynamics governed by the usual diffusion equation.

The time interval between initial and final stages of the anomalous regime may be used to estimate the burst effect during the drug release process, where the change of regime is verified. After burst, we may expect that system relaxes to the usual diffusion, which is characterized by a linear time dependence of the mean square displacement, as shown in Fig 5. For the cases shown in Fig 4, the burst is estimated in 98 min. and 150 min. for (1) and (2), respectively. It is worth to mention that this effect is desired to treat several diseases, in particular when the drug needs to be delivered rapidly to initiate the therapeutic effect[37]. Considering capsaicinoids—loaded PCL microparticles by the oral route, this behavior is very interesting for the desired therapeutic purpose. Indeed, the burst release may help to reach the effective concentration of capsaicinoids rapidly in plasma, whereas the controlled release might maintain the effective concentration of drug in plasma for a long time.

## Conclusion

We first investigated, from the formal point of view, the releasing and drug diffusion process by taking into account Eqs (4) and (5). Eq (6) represents the kinetic processes occurring on the polymer surface, which can be drug sorption or release. A particular prediction of this model was compared to experimental data obtained from in vitro dissolution of capsaicinoids-loaded PCL microparticles. We obtained a good fit for each concentration (MC3 and MC5) with the model presented above. By using this model, the mean square displacement for each case was analyzed and it was demonstrated that the Fickian diffusion governs the asymptotic limit of small and long times, as can be seen in Fig 5. The non—Fickian behavior is manifested for intermediate times. This fact leads us to conjecture that anomalous behavior of the mean square displacement may be an evidence of the burst effect, in this polymeric system. Finally, we hope that the results presented in this work are useful to investigate different scenarios characterized by drug diffusion and release.
